# Integrating Multiculturalism Into Artificial Intelligence-Assisted Programming Lessons: Examining Inter-Ethnicity Differences in Learning Expectancy, Motivation, and Effectiveness

**DOI:** 10.3389/fpsyg.2022.868698

**Published:** 2022-06-13

**Authors:** Chia-Wei Tsai, Yi-Wei Ma, Yao-Chung Chang, Ying-Hsun Lai

**Affiliations:** ^1^Department of Computer Science and Information Engineering, National Taitung University, Taitung City, Taiwan; ^2^Department of Electrical Engineering, National Taiwan University of Science and Technology, Taipei City, Taiwan

**Keywords:** multiculturalism, program education, expectancy-value theory, AI-assisted program, learning effectiveness

## Abstract

Given the current popularization of computer programming and the trends of informatization and digitization, colleges have actively responded by making programming lessons compulsory for students of all disciplines. However, students from different ethnic groups often have different learning responses to such lessons due to their respective cultural backgrounds, the environment in which they grew up, and their consideration for future employment. In this study, an AI-assisted programming module was developed and used to compare the differences between multi-ethnic college students in terms of their theoretical and actual learning expectancy, motivation, and effectiveness. The module conducted analysis through the deep learning network and examined the relevant processes that the students underwent during programming lessons, as well as the types of errors they had committed. Their learning motivation for and actual learning performance in programming were then examined based on the cognitive learning theory. The results of the experiment, which involved 96 multi-ethnic college students, indicated that the two groups had dissimilar theoretical performance in terms of their expectancy and motivation for learning programming. The indigenous students’ main concern was whether programming would affect their families or tribes, and this concern affected and was reflected in their learning outcomes. In contrast, the learning motivation and goals of Han Chinese students were driven by the cognition of the value of programming to themselves. The research findings can contribute toward the cognition and understanding of multi-ethnic students when learning computer programming and development of the appropriate teaching methods, and serve as a reference for subsequent research on integrating multiculturalism into computer programming lessons.

## Introduction

With scientific and technological developments, the application of information technology (IT) has gradually become inseparable from the various aspects of human life like food, clothing, housing, transportation, education, and entertainment. This situation is exacerbated by the latest trends of the Internet of Things (IoT) and artificial intelligence (AI), such that programming languages have become a common language and basic capability of the 21st century (Essel et al., 2017; Nouri et al., 2020). In response, countries around the world have formulated the related education policies. Bill Gates (2008) from the United States advocated computer programming as “a basic skill in the 21st century that every student should learn.” In 2016, then-President Obama emphasized that in the AI era, IT capabilities have become a new set of general skills that all citizens must possess. He also signed the “National Artificial Intelligence Research and Development Strategic Plan,” which designated computer science as a key academic field and general capability (U.S. National Science and Technology Council, 2016). Schools were also encouraged to include IT science as a subject in their foundational curriculum.

In the United Kingdom, programming was included in the curriculum in 2014. The aim was to teach children programming languages from an early age, with the requirement being the mastery of at least two such languages (Sangwin and O’Toole, 2017). In South Korea, public junior high schools introduced programming lessons in their first-year curriculum since 2018 to ensure that the students learn about IT from an early age. The purpose goes beyond teaching students how to use computers; they are taught the way computers work and about structured processes, to develop their analytical and problem-solving skills (Hsu et al., 2018). Japan announced its “Future Investment Strategies” in 2017, which incorporated programming into the curriculum of elementary and junior high school education, together with the development of teaching materials and systems for programming (Kanemune et al., 2017). The overall educational goal was to cultivate thinking in programming and solve problems through IT.

In 2019, programming lessons were officially added to the school curriculum in Republic of China (Taiwan). Elementary and junior high schools were required to train their students in programming skills and give them the foundation to acquire the relevant IT skills through computer-based learning. This way, their systematic thinking abilities are cultivated and their logical computing skills are strengthened, thereby improving their problem-solving skills, teamwork, and innovative thinking abilities through their own training capabilities (Lin, 2019; Saputro et al., 2019). Colleges have also adopted basic computer programming as a general educational course for students in all faculties, not only those in science and engineering. One targeted outcome is the search for ways of thinking through inter-disciplinary integration; another is to develop students’ non-programming abilities through training and classes in computer programming (Gong et al., 2020).

The relevant studies have pointed out that learning programming-related skills help learners improve their analytical skills, identify their problem-solving abilities, and stimulate their curiosity through the problem-solving process. Other studies showed that the process of learning programming enhances critical thinking by finding ways to think about problems. The main goal of these IT or programming lessons is not only to give trainees the basic knowledge on how to write programs, but also to train them to improve their own living conditions by reflecting on their own unique experiences, as well as identify the thinking and learning methods that best suit them through constantly learning from mistakes. This form of educational spirit and proposition in non-professional fields helps strengthen the spirit of innovation and competition (Juškevičienė et al., 2021; Lai et al., 2021).

Globally, there are many related and supporting educational course designs intended to train and cultivate in students the relevant abilities for future competitiveness and problem solving through programming. For example, Li (2018) proposed combining programming with the flipped classroom so that students cultivate self-training, and then learn the basic knowledge through self-directed learning. The students’ overall learning motivation would then be effectively enhanced. Moreover, with problems in life becoming increasingly complex, computational thinking can be used to effectively analyze complex issues. Problems can be solved with computational concepts, and humans’ problem-solving behaviors can be analyzed and understood. Computational thinking is not about making humans think like computers. Rather, it is about the full set of thinking tools needed to effectively solve complex human problems. Barr and Stephenson (2011) subdivided the capability dimension of computational thinking and introduced it into other courses (such as mathematics, science, social studies, language, and art) to examine the concept of integrating the cultivation of computational thinking abilities, as well as the corresponding goals in terms of learning behaviors.

There have also been research that applied several programming-based activities to support learners’ reading abilities, and for them to write using a programming language and engage in computational thinking. In view of the emphasis on programming lessons in the 12-year national education curriculum, many studies have highlighted the critical issues that must be overcome (Gushwa et al., 2019; Lee et al., 2019). In terms of the software and hardware in the educational environment for programming, schools need to upgrade the relevant IT equipment and set up new computer classrooms so that the learning needs of the overall school for IT education are met. In terms of teaching resources, it is crucial to cultivate more teachers in IT programming to meet the demand. It is even more necessary to introduce new concepts to students and parents so that they stop being obsessed with the expectations of traditional education, namely, prioritizing pure academic performance while ignoring the cultivation of practical and operational skills and character building. In higher education, there must be an understanding of the different ways that students from various faculties regard programming lessons. These should be introduced based on their respective professional needs so that they take to such lessons. An overall multi-professional curriculum design should be applied when cultivating and strengthening IT literacy. Emphasis should also be placed on the use of the computational thinking method for problem solving, which can double up as potential training to ready them for entering the workplace (Lye and Koh, 2018; Gürer et al., 2019; Scherer et al., 2020).

Against the trend of popularization of informatization, many indigenous families and tribes continue to live in remote areas due to the influence of history and terrain. This often results in their relative lack of economic resources and informatization level. Indigenous peoples can be further divided into tribes that dwell in the mountains or flatlands, with the former being especially lacking in their informatization level (Tzou et al., 2019; Janica, 2021). Moreover, indigenous students often lag behind in their learning performance and have a low willingness to learn because of the influence of innate environmental factors and differences in cultural cognition. Their lack in informatization skills in turn lead them to choose the service or nursing industry after graduation, and only a minority undertakes IT-related work (Lopez and Bobroff, 2019). This situation becomes a vicious cycle because there are few indigenous students who are able to bring the IT knowledge that they have learned back to their hometowns to drive the advancement of their tribe’s informatization. In the long run, the urban–rural digital gap and the indigenous tribes’ lack of informatization will keep widening.

In the spirit of diversity, equality, autonomy, and respect, the relevant government departments have been promoting education-related efforts tailored to the indigenous peoples. The aim was to establish a cultural model for indigenous education, strengthen self-identity and acceptance, and avoid the decline and loss of their unique cultures (Battiste, 2018). However, there is insufficient awareness of the cultural differences and perception of academic achievements. Many efforts wanted to take into account the tribes’ cultural characteristics and level of cultural acceptance, causing such efforts to fail to keep up with the overall trends (Eades et al., 2022). Therefore, the focus of research in multi-ethnic IT and programming education should be finding ways of using AI assisted-learning systems to effectively identify the cognitive understanding and learning performance of students from different ethnicities with regard to learning programming, and then establish an adaptive programming framework that matches the multiculturalism situation.

To further understand the relationship between learning performance and cognition of programming lessons among multi-ethnic students, an AI-assisted programming module was designed to record the students’ learning process during programming lessons, which facilitated analysis of their performance. The cognitive learning framework was used as the basis of this qualitative study to examine the impact of multi-ethnic students’ connection between self-expectancy, effort expectancy, and social value on their overall learning performance. The research findings were used to construct a theoretical foundational model for multi-ethnic education in IT programming, so as to create a multicultural learning environment that enhances students’ interest and outcomes when learning programming.

## Literature Review

### Multiculturalism and Multi-Ethnic Education

Culture is defined in anthropology as the consensus of a specific group of people regarding the sum total of their values, cognitions, codes of conduct, beliefs, customs, and material life. Multiculturalism is a critique of the emphasis on ethnic equality by liberalism without considering cultural differences, which results in the mainstream culture assimilating or rejecting marginal cultures and the latter being in a disadvantageous position. Multiculturalism advocates not only universal equality and non-assimilation, but more importantly, the recognition of one another’s cultural differences (Modood and May, 2001; Race, 2015). This is based on the spirit of multi-ethnic groups being recognized, so that the rights to their unique cultural characteristics are maintained and preserved.

Multicultural education is an indispensable key in education to create the awareness on multi-ethnic groups. The key goal is to have the participation and integration of multi-ethnic students as the basic criterion during classroom teaching activities (Ladson-Billings, 2004; Banks and Banks, 2019). In many countries around the world, research on education in science have gradually improved by taking into consideration cultural and ethnic characteristics. The perspective of multiculturalism has also been adopted to identify appropriate teaching methods and materials when teaching science to indigenous people, which involve incorporating their perspectives or acquired knowledge (Writer, 2008).

There are various approaches to multi-ethnic educational reforms internationally. These aimed at the establishment and maintenance of a multi-ethnic educational culture through the setting up of relevant systems, participation of multi-ethnic groups in educational decision-making, ensuring educational equity, and development of multicultural curriculum designs (Nikolaeva and Savvinov, 2016). Teachers bear an important responsibility in the transformation of multicultural education. A key factor is maintaining respect for and acceptance of multiculturalism, as well as establishing a curriculum that is suitable for application in the context of multi-ethnic world views and values (Blair, 2002; Denessen et al., 2007).

Some studies on the way indigenous people learn pointed out that the important reasons behind their poor academic performance can be classified into the six categories of “cultural factor,” “parents’ socioeconomic status,” “racial discrimination,” “issues pertaining to educational funding,” “issues with teaching resources,” and “issues with students’ abilities”(Duff and Li, 2009; Nikolaeva and Savvinov, 2016; Schwab, 2018). Among these, the cultural factor relates to the fact that current educational courses and teaching materials are still designed with emphasis on the mainstream culture but lack design considerations for multi-ethnic groups. Consequently, when students of marginal ethnic groups participated in the related courses, they often had a sense of psychological estrangement and uncertainty over the future. Because they did not understand how learning a course could help them realize their definition of self-worth, they often generated negative learning emotions and produced poor academic performance. The main factor behind the resolution of this situation is the establishment of multicultural education. It is only through building a multi-ethnic educational and instructional design that integrates the multi-ethnic students’ tribal experiences and values as part of the overall design that they can identify with the curriculum (Sylva et al., 2010).

The main bottleneck for multi-ethnic education is having insufficient relevant basic research. Currently, most practices in the fields of sociology and language have responded to the tribalism of multi-ethnic students. However, in terms of teaching practice in fields related to science and IT, it is difficult to respond using a culture of equality. Haberman and Post (1998) mentioned that different ethnic groups should be encouraged to design localized multicultural courses according to their cultural tendencies. Berry and Kalin (1995) also echoed that lessons on culture, language, and multiculturalism should be implemented in parallel during multicultural teaching. Doing so will ensure that various ethnic groups have equal opportunities to participate in cultural and other activities, on the premise of multiculturalism being respected and appreciated, and without their own cultural characteristics being lost.

### Information Technology Literacy and Programming Capabilities

With technological advancements, people have begun changing their living habits in response to technological changes. Physical interactions in the forms of chatting, writing letters, and shopping have gradually transformed to be based on computers and mobile phones. In the field of education, there has also been the gradual promotion of technological and digital education. Mobile computer devices allow learners to undertake learning anytime and anywhere, and the teaching and learning methods involved have gradually become increasingly reliant on computer technology (Marcum, 2002). However, such a novel and impactful way of learning is actually associated with the key factor of socioeconomic status. After all, such learning opportunities are often available only to families or regions of a relatively high socioeconomic status. In contrast, students from low-income households, marginal groups, and even indigenous tribes have difficulty accessing such learning opportunities (Loyer, 2018).

To popularize the relevant IT training in society, many colleges and tertiary institutions have begun introducing IT literacy as a general education lesson. This is so that college students gain a holistic education to respond to IT developments. Kosslyn et al. (2007) pointed out that general education not only strengthens the value of self-existence, but also encourages students to critically think about their own environments against the backdrop of the developments achieved by human civilization. They should also have the ability to deal with relevant issues and develop problem-solving skills (Serin, 2011).

Information technology literacy refers to empowering learners with the ability to use IT to pursue knowledge and solve problems, which is a means of cultivating self-thought. It can be explained in terms of intrinsic and extrinsic abilities (Ezziane, 2007; Sulisworo and Suryani, 2014). Intrinsic ability refers to the ability to think about and clarify problems, analyze the information needed in the current environment, and interpret and reorganize information into useful facts through analysis. Extrinsic ability is knowing where to find the information and how to obtain it, and using one’s self-learning ability to obtain information with which to solve the related problems. Students not only need to enrich their overall technological literacy, but should also have complete IT literacy encompassing the three dimensions of knowledge, ability, and mode of thinking and action (Brandt, 2001; Markauskaite, 2006).

Indigenous students are disadvantaged by their relative lack of living resources and cultural education, which makes it even more pertinent for them to cultivate the relevant scientific and IT literacy (Martin, 2006; Perso and Hayward, 2020). In addition to having the ability to master and control scientific and technological developments, they can use such capabilities to overcome limitations when seeking employment and enhance their employability (Pramuda et al., 2019). Moreover, they need to use their own technological thinking abilities and creativity to address the difficulties faced by the various tribes and promote their overall development based on their respective situation and conditions (Li et al., 2021).

With the popularity of AI technology in recent years, programming lessons have gradually evolved from technological literacy to technological and programming literacy. Students’ creativity can be stimulated through the process of learning programming, allowing them to build their self-learning ability, exercise their logical thinking ability, cultivate their computational thinking, and hone their problem-solving ability (Serin, 2011; Grover and Pea, 2013). With life’s problems becoming increasingly complex, computational thinking can be applied to effectively analyze complex issues. Making operational concepts process-oriented can also help solve problems and be applied to analyze and understand humans’ problem-solving behaviors.

Computational thinking is a fundamental problem-thinking skill that encompasses the entire thinking process and involves the formulation of problems and solutions. Computational thinking can be used as a standard solution for overall problems *via* computer operations or programming (Aho, 2012; Shute et al., 2017). However, it goes beyond that and is actually an essential skill for everyone, not just information or computer scientists, or even students who major in computer science. This is because it gives students of all disciplines the requisite ability to incorporate computing techniques and methods when solving problems. Barr and Stephenson (2011) subdivided the competency dimension of computational thinking and introduced it into other courses (such as mathematics, science, social studies, language, and art) to examine the concept of integrating the cultivation of computational thinking abilities, as well as the corresponding goals in terms of learning behaviors. Some research has found that combining it with the concept of computational thinking can effectively make it easier for students who are not in the professional IT field to understand the method of programming operations, thereby arousing their interest in acquiring programming skills (Barr et al., 2011; Mannila et al., 2014).

## Research Method

### Presenting the Research Model

The aim of this study was to make a theoretical assessment of multi-ethnic students’ learning expectancy and motivation for programming lessons, which affect their learning performance for the subject. The cognitive development theory was used as the basis to describe the process of their knowledge acquisition, and their performance at and changes during information processing. It includes the following periods: sensory action, pre-operation thinking, specific operation, and formal operation (Gopnik and Wellman, 1994; Huitt and Hummel, 2003). Cognitive growth has different effects on students’ various abilities. Cognition must be the building block of self-learning and the formation of self-cognition. The experience of others can only serve as reference or past anecdotes during learners’ exploration process. According to research, there are some adults who have yet to reach the formal operation period. They are unable to achieve that stage because the conditions when they were growing up prevented them from learning about, or allowed them to avoid contact with, new circumstances and objects. This situation is also more likely to occur during the learning situation of students from marginal cultures. This was a phenomenon that needed evaluation in this study because it affects the teaching of multicultural students.

Educational psychologists have pointed out that different people have dissimilar learning motivations and strategies. For example, some students prefer to find the answers by themselves, whereas others want their teachers to give them prompts so that they can solve the problems quickly. Their self-learning expectancy and motivation affect the overall learning factors. Learners can improve their overall learning efficiency by changing their learning motivation, which then enhances their learning effectiveness. Learning motivation can be divided into physiological and psychological motivation. Physiological motivation is the primordial need that drives one to engage in learning behaviors; psychological motivation is based on self-will, motivation for achievement, and expectancy. Research on learning motivation is predominantly focused on analyzing psychological motivation. Many studies have pointed out that when students feel that the relevant knowledge and cognition are of value or can help them gain a sense of achievement with their group, their intrinsic motivation is raised. This increases their positive learning attitudes, which include level of commitment, awareness of self-worth, satisfaction level with the teaching, and positive feedback between teachers and students.

Pintrich et al. (1993) first proposed the theoretical model of expectancy and motivation, and conducted research on learning through the three main motivations of value, expectancy, and emotion. Value motivation refers to the value obtained by learners’ interaction with the relevant learning objectives, and is their reason for learning. Expectancy motivation refers to the ability that learners expect to gain after learning, or the expectancy of learning success. Emotional motivation refers to how much learners like the behavior of learning, or their emotional response during learning. MacIntyre and Blackie (2012) introduced another category of learning motivation in the form of volitional motivation, which refers to whether learners persist and implement the learning behaviors acquired during the learning process. Indigenous students often live adjacent to their fellow tribespeople and they learn the appropriate behaviors and actions by observing the latter. Although this environmental element is not affected by the teaching materials that the students come across, it produces in them the phenomena of self-motivation and -adjustment. People construct the corresponding educational methods based on their observations and through self-learning (Bandura, 1989). The overall perspective also has a relative impact on indigenous students’ expectancy through organizational expectancy and industrial structure, forming a tripartite mode of interaction.

The social cognitive career theory (SCCT), which was derived from this form of social cognitive theory, also affects learners’ career choices. During research on students’ interests in different fields, additional considerations are given to the disguised forms of learning interests, achievement abilities, and goal selection (Lent et al., 2002). In this study, the SCCT was used to examine the impact of indigenous students’ cognitive motivation when they learn programming.

### The Module Used for Analysis

The module developed in this study examined the students’ situation when they learn programming in order to analyze their learning performance. In this study, Python 3.0 was adopted as the standard programming language for learning and used to collect data during programming lessons. Typically, when students learn to write programs, they would conduct functional validation after their programs have been compiled by a compiler, and then perform unit testing to ensure that what they have learned is executed correctly.

When learning programming, learners will encounter various erroneous situations during the compiling stage. These include syntax or logic errors including writing errors for the program’s functional module and failures in the definition of variables, which prevent the compiler from completing its execution. Its platform mainly captures and compiles records of failures. After obtaining the compilation of recorded failures, a textual search was performed and the erroneous information was recorded. Next, text mining was used to search for the relevant syntax error in the “substitute search word,” before comparing the possible errors for that word generated from the database of line numbers and keywords, and categorizing those errors. The other check was to introduce an empty function set to generate a data defect, so that the detection of its internal error and address could produce the empty data error function before comparing its line number and string characters.

The unit test, which is performed after the basic compilation is completed, analyzes the Python code through Pylint. Its analytical suite uses the PEP 8 coding style for detection, and lists whether the codes being detected and validated have violated any PEP 8 specifications or contained common errors. The data being reported are labeled as C and W for convention and warning, respectively. There are six levels of Pylint results in total. After using Pylint to validate the results of executing the codes, information of the relevant results and line numbers are recorded. Finally, these are matched with the coding coverage to predict the program’s operating or functional errors. Detection of the coding coverage is mainly to confirm whether the overall codes allow the coding process to be executed completely and for purpose of memory validation. These form the basis for further estimating whether the codes will generate more errors.

There are many tools for detecting coding errors that use error location methods to identify the corresponding errors. In this study, the coding coverage and Pylint detection results were used to predict the programming results. The module compares the results with pre-loaded correct templates, and then use path analysis to predict and compare the two execution paths, and determine the coverage and number of warning lines. After deconstructing the entire program, it will determine the status of the results. The module records the user’s overall time spent on programming practice, completion of the functions, number of compilations made, corresponding correction data, data coverage, and program check line count.

In each lesson, the students were given 30 min to write a functional program for the IoT. Items with syntax errors and the related compilation time and type of errors were recorded through the program’s compiler interface. Items with logic errors and warnings were analyzed using the program’s logging function and Pylint.

## Research Findings

In this study, 96 students who studied in information engineering were invited as participants, with 62 and 34 being Han Chinese and indigenous people, in information programming courses. The subjects were aged between 18 and 22, with a male to female ratio of 80–16, and all had basic information programming skills. Before each class, teachers will introduce the basic functions of this course to ensure that each participant has basic ability and knowledge. Data collection and survey with cognitive questionnaires were conducted over six programming lessons. The purpose was to examine the relationship between the expectancy of self-worth of students from multi-ethnic groups and their learning motivation and process, as well as understand their performance when learning programming. PLS-SEM was used to analyze the relevant data because of the small sample size (Hair et al., 2017). Given that there were six indicators in this study, 60 samples was the minimum number required to meet the evaluation criteria according to the 10 times rule (Chin and Newsted, 1999).

### Analysis of the Results Recorded by the Module

**FIGURE 1:**
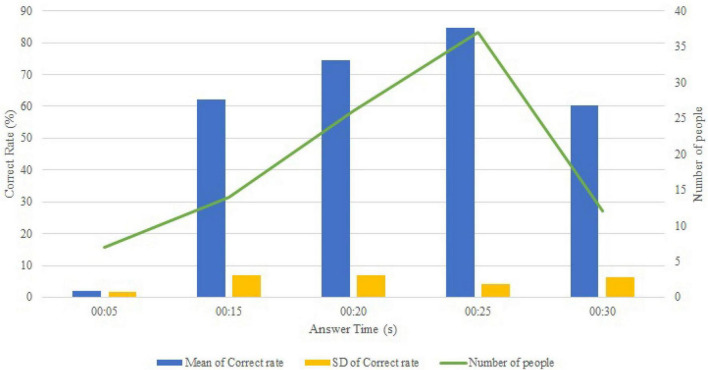
Statistics on correct answer rates.

The module recorded each student’s process of learning program, their correct answers (error rate), answering time, and the number of syntax errors, logic errors, and warnings. First, the correct rates for completing the programming task at various response times were analyzed shown as Figure 1. The correct rate at 5 min was only 2%. This was because the students in this category had given up on the task at the early stage and simply made their submissions. The majority of the students completed their task in approximately 20–25 min, with the correct rate being approximately 71–85%. When the response time reached 30 min, the correct rate dropped to 60% and the standard deviation (SD) increased to approximately 12%. Upon checking, this was determined to be caused by those students who had not completed the task but continued working on it.

**FIGURE 2:**
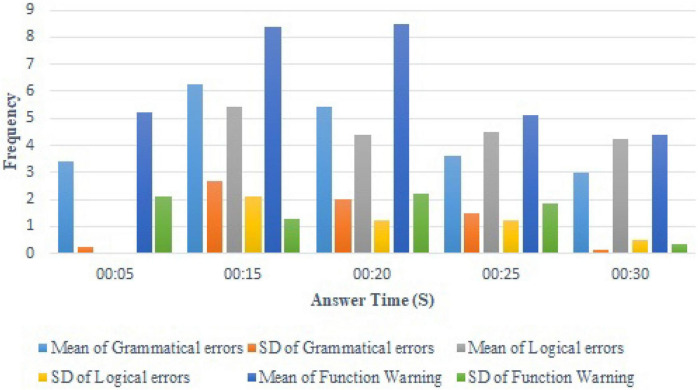
Statistics on the detailed analysis of recorded errors.

A detailed analysis was made of the recorded errors corresponding to the various time points and the statistical results are shown in Figure 2. The number of syntax errors gradually decreased with time, but the number of logic errors did not decrease significantly. There were more warnings at the 15–20-min period, but these gradually decreased at the 25–30-min period. The SD decreased significantly at the 30-min time point. A possible reason was the students making a final attempt to check with one another before the allocated time was up.

### Learning Motivation and Effectiveness of Han Chinese Versus Indigenous Students

The learning motivation and performance of Han Chinese and indigenous students were analyzed in terms of values, expectancy, emotions, and social expectations. SmartPLS was used to analyze the data from the questionnaires. The first step was to eliminate data for those questionnaire items with factor loadings below 0.7. The expectancy cognition will be 0.684 in Cronbach’s alpha and CR will be 0.524 and social expectancy will be 0.425 in Cronbach’s alpha and CR will be 0.337. However, because this item of poor validity will affect the overall effect, it will be removed. After analysis, the Cronbach’s alpha and rho_A of all the items were greater than 0.7. Although the composite reliability (CR) for the item was slightly below 0.7, it was still within the acceptable range (Fornell and Larcker, 1981). The average variance extracted (AVE) of all the items was greater than 0.5, which was in line with the convergent validity for variations. The heterotrait-monotrait ratio (HTMT) was used to determine the discriminant validity of the various facets. All the facets met the requirement of discriminant validity < 0.9 (Henseler et al., 2016).

**FIGURE 3:**
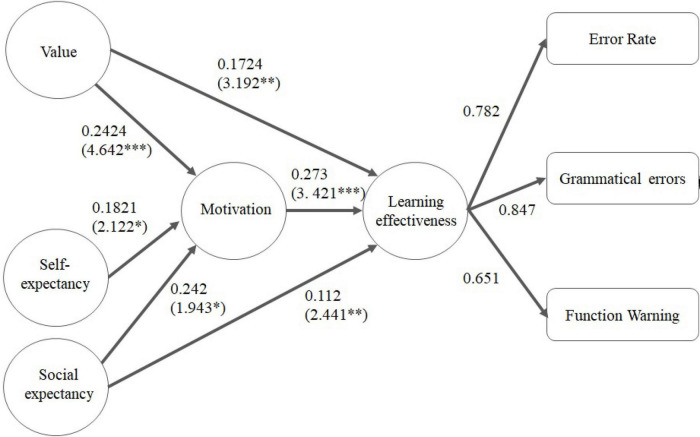
Impact of Han Chinese students’ learning motivation on their learning performance.

**FIGURE 4:**
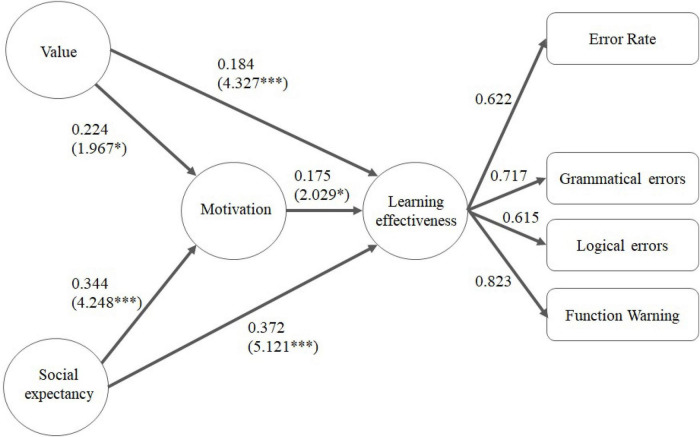
Impact of students’ learning motivation on their learning performance.

Next, bootstrapping (with 5,000 iterations) was used to calculate the related *t*-value, *p*-value, and *R*^2^ value. The overall results are shown in Figures 3, 4, which indicate that their emotional cognition did not correspond to the test results for learning motivation. In the figures, ^**^ and ^***^ means the * *t* > 1.96 (*p* < 0.05), ^**^ *t* > 2.58 (*p* ≤ 0.01), and ^***^ *t* > 3.29 (*p* ≤ 0.001). There were also differences between the Han Chinese and indigenous students in terms of their expectancy cognition. For Han Chinese students, the relevant indicators of learning motivation are value cognition, self-expectancy, and social expectancy. Especially in the value cognition, the *t* value of the influence on motivation is 4.642, which is more significant than self-expectation and social painting expectations; the path coefficient is 0.2424, which is similar to the social expectation of 0.242. In learning effectiveness, values, learning motivation and social expectancy also showed significant correlations. In the learning effectiveness indicators, the factor loading of error rate, grammatical errors and function warnings are higher than 0.6, while those of answering time, logical errors and coverage ratio are lower than 0.6 and are excluded. For indigenous students, the relevant indicators of learning motivation are value cognition and social expectancy. Specifically, their self-expectancy regarding IT programming did not significantly correlate with their learning motivation. The impacts of social expectancy and value cognition on learning motivation varied among the Han Chinese students, for whom the impact of social expectations was not as strong as that for the indigenous students. For the latter, there were stronger correlations between social expectations and their learning motivation and performance. In the learning effectiveness indicators, the factor loading of error rate, grammatical errors, logical errors, and function warnings are higher than 0.6, while those of answering time and coverage ratio are lower than 0.6 and are excluded. According to the experimental results of Further discussion on why the factor loading is too low in the answering time. According to the experimental results, it can be found that the index value cannot reflect the learning effect obviously because some students who answered the question too short or gave up waiting for the answer for a long time, it can be found that the index value cannot reflect the learning effectiveness obviously because some students who answered the question too short or gave up waiting for the answer for a long time. The coverage ratio speculation may not be correctly reflected in the learning effectiveness due to the occurrence of plagiarism in each stage.

## Research Limitations and Discussion

In this study, a deconstruction programming module was developed to analyze and examine the learning performance of multi-ethnic students when they attend programming lessons. In this study limitations, the object of this study does not fully explain the value perception of all aboriginal students in learning. The research process was explained through the recruitment instructions. It cannot be denied that for some participants, the process of learning programming might possibly have been affected by the research (for example, delay in submission of task, checking with other participants and asking them questions, and the success rate). Although four weeks of training on basic computing abilities were conducted for the participants at the early stage of the study, the impact of other factors such as the cognitive load of IoT module circuit on their learning effectiveness cannot be completely ruled out.

i. In terms of learning motivation, both groups of students showed correlations between their value cognition and social expectations, although social expectations had a stronger impact on the indigenous students’ learning motivation than that of the Han Chinese students. The indigenous students were more concerned about whether the learning of IT programming would affect their families or tribes, and this concern was also reflected in terms of the impact on their learning outcomes. Interviews with some of the indigenous participants revealed that they were aware of the positive impacts of IT on their family’s finances, and that their tribes will have a better future with development in informatization. However, it is worth noting that when their tribe or family had imposed a negative expectancy on them, it would cause them to give up easily or even disrupt their learning in IT.
ii. There was a correlation between the Han Chinese students’ value and expectancy cognition and their learning motivation. This was not the case for indigenous students. However, the learning motivation of both groups of students correlated with their value cognition. During the interviews, some indigenous students concurred that IT programming could lead to job opportunities and increase in self-worth. However, they lacked expectancy of their future employment. The main reasons were that indigenous students are predominantly employed in the service industry upon graduation, and job opportunities in the IT industry are lacking in their tribes. Therefore, through the popularization of information from the indigenous tribes, it will be more able to help the indigenous students to understand the value of information education.
iii. The impact of learning motivation on their learning performance was different for both groups of students. For the Han Chinese students, their learning motivation affected their overall performance in IT learning; for the indigenous students, even though they might have learning motivation, the presence of economic or other factors ultimately affected their learning performance. In contrast, there were cases where their learning motivation was not obvious but their learning performance ended up being excellent. For future studies, the experimental process can be implemented using a general and foundational learning course for programming instead and add relevant culturally responsive teaching methods to help enhance the motivation of indigenous people to learn programming education.

## Data Availability Statement

The raw data supporting the conclusions of this article will be made available by the authors, without undue reservation.

## Author Contributions

C-WT was in charge of the course program teaching and research process implementation. Y-WM designed and developed the AI-assisted platform. Y-CC was responsible for data analysis and discussion. Y-HL proposed the overall research model and data-oriented research design. All authors contributed to the article and approved the submitted version.

## Conflict of Interest

The authors declare that the research was conducted in the absence of any commercial or financial relationships that could be construed as a potential conflict of interest.

## Publisher’s Note

All claims expressed in this article are solely those of the authors and do not necessarily represent those of their affiliated organizations, or those of the publisher, the editors and the reviewers. Any product that may be evaluated in this article, or claim that may be made by its manufacturer, is not guaranteed or endorsed by the publisher.

## References

[B1] Aho A. V. (2012). Computation and computational thinking. *Comput. J.* 55 832–835. 10.1093/comjnl/bxs074

[B2] Bandura A. (1989). Regulation of cognitive processes through perceived self-efficacy. *Dev. Psychol.* 25:729. 10.1037/0012-1649.25.5.729

[B3] Banks J. A. Banks C. A. M. (2019). *Multicultural Education: Issues and Perspectives.* John Wiley & Sons.

[B4] Barr D. Harrison J. Conery L. (2011). Computational thinking: a digital age skill for everyone. *Learn. Lead. Technol.* 38 20–23.

[B5] Barr V. Stephenson C. (2011). Bringing computational thinking to K-12: what is involved and what is the role of the computer science education community? *ACM Inroads* 2 48–54.

[B6] Battiste M. (2018). “Reconciling indigenous knowledge in education: promises, possibilities, and imperatives,” in *Dissident Knowledge in Higher Education*, eds Spooner M. McNinch J. (Regina, SK: University of Regina Press), 123–148.

[B7] Berry J. W. Kalin R. (1995). Multicultural and ethnic attitudes in Canada: an overview of the 1991 national survey. *Can. J. Behav. Sci. Rev. Can. Des Sci. Du Comport.* 27:301. 10.1037/0008-400X.27.3.301

[B8] Blair M. (2002). Effective school leadership: the multi-ethnic context. *Br. J. Soc. Educ.* 23 179–191. 10.1080/01425690220137701

[B9] Brandt D. S. (2001). Information technology literacy: task knowledge and mental models. *Libr. Trends* 50, 73–86.

[B10] Chin W. W. Newsted P. R. (1999). Structural equation modeling analysis with small samples using partial least squares. *Statist. Strat. Small Sample Res.* 1 307–341.

[B11] Denessen E. Bakker J. Gierveld M. (2007). Multi-ethnic schools’ parental involvement policies and practices. *Sch. Community J*. 17, 27–44.

[B12] Duff P. A. Li D. (2009). Indigenous, minority, and heritage language education in Canada: policies, contexts, and issues. *Can. Mod. Lang. Rev.* 66 1–8. 10.3138/cmlr.66.1.001

[B13] Eades O. Toombs M. R. Cinelli R. Easton C. Hampton R. Nicholson G. C. (2022). The path to eldership: results from a contemporary indigenous Australian community. *Gerontologist* 62 607–615. 10.1093/geront/gnab062 33978151

[B14] Essel H. B. Nunoo F. K. N. Ahiaklo-Kuz N. A. Y. (2017). Development of an integrated art and visual programming framework for ghanaian basic schools based on a 21st century skill deficiency diagnostic on two basic school subjects. *J. Educ. Hum. Dev.* 6 89–98. 10.15640/jehd.v6n4a10

[B15] Ezziane Z. (2007). Information technology literacy: implications on teaching and learning. *J. Educ. Technol. Soc.* 10 175–191.

[B16] Fornell C. Larcker D. F. (1981). Structural equation models with unobservable variables and measurement error: algebra and statistics. *J. Mark. Res*. 18, 382–388.

[B17] Gates B. (2008). Strengthening American competitiveness for the 21st century. *Yearbook Natl. Soc. Study Educ.* 107 95–98. 10.1111/j.1744-7984.2008.00173_1.x

[B18] Gong D. Yang H. H. Cai J. (2020). Exploring the key influencing factors on college students’ computational thinking skills through flipped-classroom instruction. *Int. J. Educ. Technol. Higher Educ.* 17 1–13. 10.1186/s41239-020-00196-0

[B19] Gopnik A. Wellman H. M. (1994). *The Theory. In an Earlier Version Of This Chapter Was Presented At The Society For Research In Child Development Meeting, 1991.* Cambridge: Cambridge University Press.

[B20] Grover S. Pea R. (2013). Computational thinking in K–12: a review of the state of the field. *Educ. Res.* 42 38–43. 10.3102/0013189X12463051

[B21] Gürer M. D. Cetin I. Top E. (2019). Factors affecting students’ attitudes toward computer programming. *Inf. Educ*. 18, 281–296.

[B22] Gushwa M. Bernier J. Robinson D. (2019). Advancing child sexual abuse prevention in schools: an exploration of the effectiveness of the enough! Online training program for K-12 teachers. *J. Child Sexual Abuse* 28 144–159. 10.1080/10538712.2018.1477000 29792582

[B23] Haberman M. Post L. (1998). Teachers for multicultural schools: the power of selection. *Theory Pract.* 37 96–104. 10.1016/j.jtbi.2017.06.032 28663015

[B24] Hair J. F. Hult G. T. M. Ringle C. M. Sarstedt M. Thiele K. O. (2017). Mirror, mirror on the wall: a comparative evaluation of composite-based structural equation modeling methods. *J. Acad. Mark. Sci.* 45 616–632. 10.1007/s11747-017-0517-x

[B25] Henseler J. Hubona G. Ray P. A. (2016). Using PLS path modeling in new technology research: updated guidelines. *Ind. Manage. Data Syst*. 116, 2–20. 10.1108/IMDS-09-2015-0382

[B26] Hsu T. C. Chang S. C. Hung Y. T. (2018). How to learn and how to teach computational thinking: Suggestions based on a review of the literature. *Comput. Educ.* 126 296–310. 10.1016/j.compedu.2018.07.004

[B27] Huitt W. Hummel J. (2003). Piaget’s theory of cognitive development. *Educ. Psychol. Interac*t. 3, 1–5.

[B28] Janica E. V. (2021). “Math & crafts, educational activities: 400 indigenous kids learning math from engineers and scientists,” in *Proceeding of the 2021 IEEE Integrated STEM Education Conference (ISEC)*, (IEEE), 45–50. 10.1109/ISEC52395.2021.9764076

[B29] Juškevičienė A. Stupurienė G. Jevsikova T. (2021). Computational thinking development through physical computing activities in STEAM education. *Comput. Appl. Eng. Educ.* 29 175–190. 10.1002/cae.22365

[B30] Kanemune S. Shirai S. Tani S. (2017). Informatics and programming education at primary and secondary schools in Japan. *Olymp. Inform.* 11 143–150. 10.15388/ioi.2017.11

[B31] Kosslyn S. M. Liu D. R. Menand L. Petersen R. A. Pilbeam D. R. Simmons A. (2007). Report of the task force on general education. *Retrieved November* 12:2013.

[B32] Ladson-Billings G. (2004). New directions in multicultural education. *Handbook Res. Multicult. Educ.* 2 50–65.

[B33] Lai Y. H. Chen S. Y. Lai C. F. Chang Y. C. Su Y. S. (2021). Study on enhancing AIoT computational thinking skills by plot image-based VR. *Interact. Learn. Environ.* 29 482–495. 10.1080/10494820.2019.1580750

[B34] Lee J. Finn J. Liu X. (2019). Time-indexed effect size for educational research and evaluation: reinterpreting program effects and achievement gaps in K–12 reading and math. *J. Exp. Educ.* 87 193–213. 10.1080/00220973.2017.1409183

[B35] Lent R. W. Brown S. D. Hackett G. (2002). Social cognitive career theory. *Career Choice Dev.* 4 255–311.

[B36] Li J. Brar A. Roihan N. (2021). The use of digital technology to enhance language and literacy skills for Indigenous people: a systematic literature review. *Comput. Educ. Open* 2:100035. 10.1016/j.caeo.2021.100035

[B37] Li Y. (2018). Current problems with the prerequisites for flipped classroom teaching—a case study in a university in Northwest China. *Smart Learn. Environ.* 5 1–23. 10.1186/s40561-018-0051-4

[B38] Lin Y. T. (2019). Impacts of a flipped classroom with a smart learning diagnosis system on students’ learning performance, perception, and problem solving ability in a software engineering course. *Comput. Hum. Behav.* 95 187–196. 10.1016/j.chb.2018.11.036

[B39] Lopez J. H. Bobroff K. L. (2019). Rooted indigenous core values: culturally appropriate curriculum and methods for civic education reflective of native american culture and learning styles. *Multicult. Perspect.* 21 119–126. 10.1080/15210960.2019.1606640

[B40] Loyer J. (2018). *Indigenous Information Literacy: Nêhiyaw Kinship Enabling Self-Care in Research*. Sacramento, CA: Library Juice Press.

[B41] Lye S. Y. Koh J. H. L. (2018). *Case Studies Of Elementary Children’s Engagement In Computational Thinking Through Scratch Programming Computational Thinking in the STEM Disciplines.* Cham: Springer, 227–251. 10.1007/978-3-319-93566-9_12

[B42] MacIntyre P. D. Blackie R. A. (2012). Action control, motivated strategies, and integrative motivation as predictors of language learning affect and the intention to continue learning French. *System* 40 533–543. 10.1016/j.system.2012.10.014

[B43] Mannila L. Dagiene V. Demo B. Grgurina N. Mirolo C. Rolandsson L. (2014). “Computational thinking in K-9 education,” in *Proceedings of the Working Group Reports of the 2014 on Innovation & Technology in Computer Science Education Conference*, 1–29. 10.1145/2713609.2713610

[B44] Marcum J. W. (2002). Rethinking information literacy. *Library Quart.* 72 1–26. 10.1086/603335

[B45] Markauskaite L. (2006). Towards an integrated analytical framework of information and communications technology literacy: from intended to implemented and achieved dimensions. *Inform. Res. Int. Electr. J.* 11:n3.

[B46] Martin A. J. (2006). A motivational psychology for the education of indigenous Australian students. *Austr. J. Indig. Educ.* 35 30–43. 10.1017/S1326011100004142

[B47] Modood T. May S. (2001). Multiculturalism and education in britain: an internally contested debate. *Int. J. Educ. Res.* 35 305–317. 10.1016/S0883-0355(01)00026-X 26133619

[B48] Nikolaeva A. D. Savvinov V. M. (2016). Multi-ethnic school in the russian federation: the preconditions of formation and development (a case study of a national region). *Int. Electr. J. Math. Educ.* 11 3405–3414.

[B49] Nouri J. Zhang L. Mannila L. Norén E. (2020). Development of computational thinking, digital competence and 21st century skills when learning programming in K-9. *Educ. Inquiry* 11 1–17. 10.1080/20004508.2019.1627844

[B50] Perso T. Hayward C. (2020). *Teaching Indigenous Students: Cultural Awareness and Classroom Strategies for Improving Learning Outcomes*. London: Routledge. 10.4324/9781003117728

[B51] Pintrich P. R. Smith D. A. Garcia T. McKeachie W. J. (1993). Reliability and predictive validity of the motivated strategies for learning questionnaire (MSLQ). *Educ. Psychol. Measure.* 53 801–813. 10.1177/0013164493053003024

[B52] Pramuda A. Kuswanto H. Hadiati S. (2019). Effect of real-time physics organizer based smartphone and indigenous technology to students’ scientific literacy viewed from gender differences. *Int. J. Instruct.* 12 253–270. 10.29333/iji.2019.12316a

[B53] Race R. (2015). *Multiculturalism and Education*. London: Bloomsbury Publishing.

[B54] Sangwin C. J. O’Toole C. (2017). Computer programming in the UK undergraduate mathematics curriculum. *Int. J. Math. Educ. Sci. Technol.* 48 1133–1152. 10.1080/0020739X.2017.1315186

[B55] Saputro A. D. Atun S. Wilujeng I. (2019). The impact of problem solving instruction on academic achievement and science process skills among prospective elementary teachers. *Ilkogretim Online* 18:2.

[B56] Scherer R. Siddiq F. Viveros B. S. (2020). A meta-analysis of teaching and learning computer programming: effective instructional approaches and conditions. *Comput. Hum. Behav.* 109:106349. 10.1016/j.chb.2020.106349

[B57] Schwab R. (2018). *Why Only One In Three? The Complex Reasons For Low Indigenous School Retention.* Canberra, ACT: Centre for Aboriginal Economic Policy Research (CAEPR), The Australian National University.

[B58] Serin O. (2011). The effects of the computer-based instruction on the achievement and problem solving skills of the science and technology students. *Turkish Online J. Educ. Technol. TOJET* 10 183–201.

[B59] Shute V. J. Sun C. Asbell-Clarke J. (2017). Demystifying computational thinking. *Educ. Res. Rev.* 22 142–158. 10.1016/j.edurev.2017.09.003

[B60] Sulisworo D. Suryani F. (2014). The effect of cooperative learning, motivation and information technology literacy to achievement. *Int. J. Learn. Dev.* 4 58–64. 10.5296/ijld.v4i2.4908

[B61] Sylva T. Chinn P. Kinoshita C. (2010). A culturally relevant agricultural and environmental course for K–12 teachers in hawaii. *J. Natl. Res. Life Sci. Educ.* 39 10–14. 10.4195/jnrlse.2008.0040k 14966996

[B62] Tzou C. Suárez E. Bell P. LaBonte D. Starks E. Bang M. (2019). Storywork in STEM-art: making, materiality and robotics within everyday acts of indigenous presence and resurgence. *Cognition and Instruction* 37 306–326. 10.1080/07370008.2019.1624547

[B63] U.S. National Science and Technology Council (2016). *National Artificial Intelligence Research and Development Strategic Plan.* Washington DC: Networking and Information Technology Research and Development Subcommittee.

[B64] Writer J. H. (2008). Unmasking, exposing, and confronting: critical race theory, tribal critical race theory and multicultural education. *Int. J. Multicult. Educ*. 10, 1–15. 10.18251/ijme.v10i2.137

