# Supplementary material for: Integrating Multiculturalism Into Artificial Intelligence-Assisted Programming Lessons: Examining Inter-Ethnicity Differences in Learning Expectancy, Motivation, and Effectiveness
Source: Front Psychol. 2022 Jun 13;13:868698. doi: 10.3389/fpsyg.2022.868698 (PMC9234485; doi:10.3389/fpsyg.2022.868698)
Supplement: Supplementary file 1 [file Data_Sheet_1.pdf]

# *Supplementary Material*

## Supplementary Figures & Tables

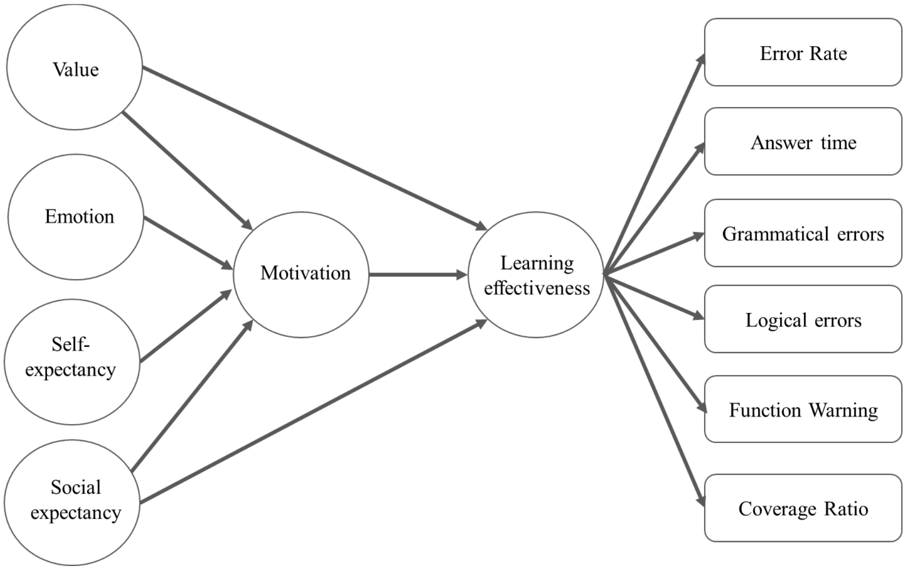


**Supplementary Figure 1**. Research model

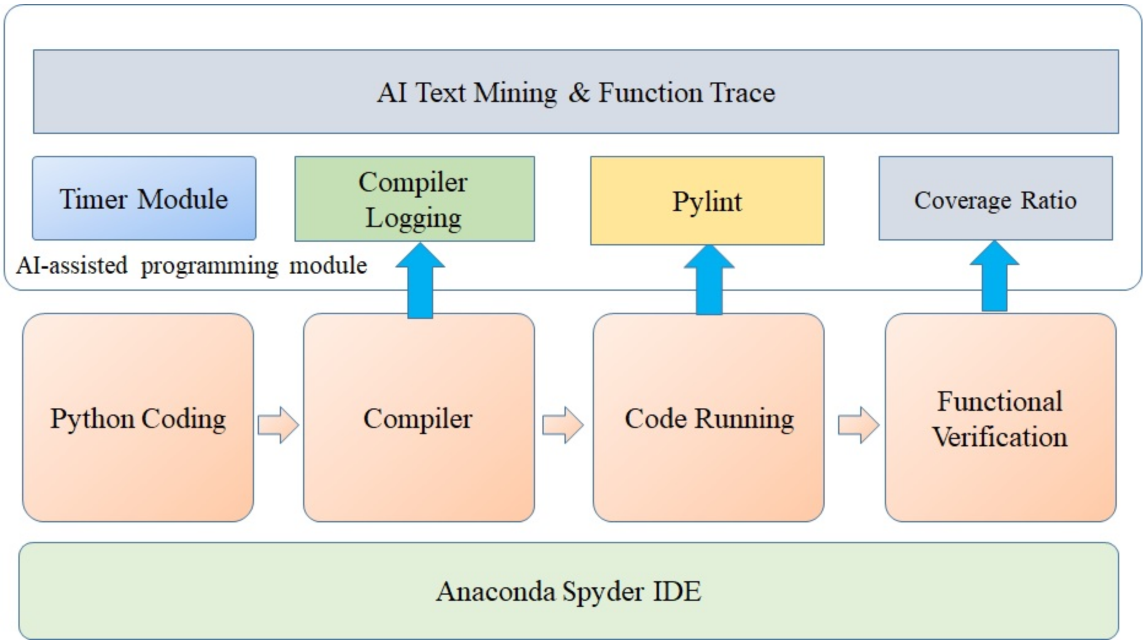


**Supplementary Figure 2**. Architecture of the developed module

**Supplementary Table 1. Reliability and validity testing of the facets**

| | Cronbach's Alpha | rho_A | CR | AVE |
|---|---|---|---|---|
| Value cognition | 0.713 | 0.724 | 0.682 | 0.524 |
| Expectancy cognition | 0.752 | 0.725 | 0.812 | 0.651 |
| Emotional cognition | 0.751 | 0.812 | 0.802 | 0.753 |
| Social expectations | 0.722 | 0.723 | 0.681 | 0.520 |
| Learning motivation | 0.768 | 0.852 | 0.714 | 0.564 |
| Learning effectiveness | 0.823 | 0.892 | 0.845 | 0.752 |

**Supplementary Table 2. HTMT of the facets**

| | Value cognition | Expectancy cognition | Emotional cognition | Social expectations | Learning motivation | Learning performance |
|---|---|---|---|---|---|---|
| **Value cognition** | | | | | | |
| **Expectancy cognition** | 0.751 | | | | | |
| **Emotional cognition** | 0.622 | 0.512 | | | | |
| **Social expectations** | 0.681 | 0.558 | 0.428 | | | |
| **Learning motivation** | 0.645 | 0.542 | 0.528 | 0.745 | | |
| **Learning effectiveness** | 0.741 | 0.582 | 0.314 | 0.641 | 0.514 | |
